# Identification of Two bZIP Transcription Factors Interacting with the Promoter of Soybean Rubisco Activase Gene (*GmRCA*α)

**DOI:** 10.3389/fpls.2016.00628

**Published:** 2016-05-17

**Authors:** Jinyu Zhang, Hongyang Du, Maoni Chao, Zhitong Yin, Hui Yang, Yakai Li, Fang Huang, Deyue Yu

**Affiliations:** ^1^National Center for Soybean Improvement, National Key Laboratory of Crop Genetics and Germplasm Enhancement, Nanjing Agricultural UniversityNanjing, China; ^2^Henan Collaborative Innovation Center of Modern Biological Breeding, Henan Institute of Science and TechnologyXinxiang, China; ^3^Jiangsu Provincial Key Laboratory of Crop Genetics and Physiology, Yangzhou UniversityYangzhou, China

**Keywords:** Rubisco activase, promoter, basic leucine zipper, soybean, transcription factor

## Abstract

Rubisco activase (RCA), a key photosynthetic protein, catalyses the activation of Rubisco and thus plays an important role in photosynthesis. Although the *RCA* gene has been characterized in a variety of species, the molecular mechanism regulating its transcription remains unclear. Our previous studies on *RCA* gene expression in soybean suggested that expression of this gene is regulated by *trans*-acting factors. In the present study, we verified activity of the *GmRCA*α promoter in both soybean and *Arabidopsis* and used a yeast one-hybrid (Y1H) system for screening a leaf cDNA expression library to identify transcription factors (TFs) interacting with the *GmRCA*α promoter. Four basic leucine zipper (bZIP) TFs, GmbZIP04g, GmbZIP07g, GmbZIP1, and GmbZIP71, were isolated, and GmbZIP04g and GmbZIP07g were confirmed as able to bind to a 21-nt G-box-containing sequence. Additionally, the expression patterns of *GmbZIP04g, GmbZIp07g*, and *GmRCA*α were analyzed in response to abiotic stresses and during a 24-h period. Our study will help to advance elucidation of the network regulating *GmRCA*α transcription.

## Introduction

Ribulose-1,5-bisphosphate carboxylase/oxygenase (Rubisco), an abundant and important enzyme in plants, catalyses the photosynthetic assimilation of atmospheric CO_2_ and photorespiratory carbon oxidation (Spreitzer and Salvucci, 2002). In higher plants, Rubisco is a hexadecameric complex composed of eight large (RbcL) and eight small subunits (RbcS), and its activity *in vivo* is known to be regulated by different mechanisms (Stec, 2012). Previous studies have shown that the activation and maintenance of Rubisco catalytic activity require the continued action of Rubisco activase (RCA), an ATPase associated with a variety of cellular activities (AAA+) protein (Andrews et al., 1995; Portis, 2003). RCA is a nuclear-encoded chloroplast protein that functions as a molecular chaperone and activates Rubisco by removing various inhibitory sugar phosphates in an ATP-dependent reaction (Portis, 1990; Dejimenez et al., 1995). In addition, the expression level of *RCA* mRNA has been correlated with Rubisco activity, photosynthetic rates, seed/grain yield, and chlorophyll fluorescence parameters, suggesting its potential applicability in breeding for enhancing soybean productivity (Yin et al., 2010, 2014; Chao et al., 2014).

In higher plants, the *RCA* gene is mainly expressed in photosynthetic tissues and is developmentally regulated by both light and leaf age (Watillon et al., 1993; Jiang et al., 2013; Bayramov and Guliyev, 2014; Chao et al., 2014). Levels of *RCA* mRNA exhibit cyclic variations during the day/night period in tomato (Martinocatt and Ort, 1992), apple (Watillon et al., 1993), rice (To et al., 1999), potato (Jiang et al., 2013), soybean (Chao et al., 2014), maize (Yin et al., 2014), and *Arabidopsis* (Liu et al., 1996). Moreover, RCA expression at the mRNA and protein levels is reported to be affected by a variety factors, including abiotic stress such as ozone (Pelloux et al., 2001; Demirevska-Kepova et al., 2005), drought (Pelloux et al., 2001; Bota et al., 2004; Ji et al., 2012), UV-B light (Liu et al., 2002), heat (Demirevska-Kepova et al., 2005; DeRidder and Salvucci, 2007; Scafaro et al., 2010), and NaCl (Bayramov and Guliyev, 2014). It has been reported that the RCA protein can also be regulated by *Manduca sexta* (a specialist herbivore of *Nicotiana attenuata*) attack and that RCA has a function in regulating JA signaling (Giri et al., 2006; Walia et al., 2007; Mitra and Baldwin, 2008, 2014; Shan et al., 2011; Attaran et al., 2014). RCA is also regulated in response to ABA treatment (Fukayama et al., 2010; Zhu et al., 2010).

Gene expression is regulated both quantitatively and qualitatively by specific sequences upstream of a gene’s coding region, commonly known as the promoter, which contains multiple *cis*-regulatory elements (Mitsuhara et al., 1996). Indeed, interaction between *cis*-elements and transcription factors (TFs) has a critical role in regulating transcription via the coordinated activation or repression of the target (Ptashne, 2005; Wehner et al., 2011). Although the *RCA* gene has been characterized in a number of species, to date, its promoter has only been studied in spinach (Orozco and Ogren, 1993), *Arabidopsis* (Liu et al., 1996), potato (Qu et al., 2011), rice (Yang et al., 2012), and soybean (Chao et al., 2014), and the molecular mechanism by which the *RCA* promoter is regulated remains to be clarified. It has been shown in rice that nuclear proteins bind specifically to the *RCA* gene promoter (Yang et al., 2012). In soybean, previous studies have shown that expression of *GmRCA*β is controlled by a combination of both *cis*-acting and *trans*-acting expression quantitative trait loci (eQTLs) (Chao et al., 2014), with *GmRCA*α gene expression being mainly regulated by *trans*-acting factors (Yin et al., 2010). However, little is known about the specific proteins interacting with the *RCA* promoter.

Most plants contain two closely related isoforms of RCA, an α-isoform of 46–48 kDa and a β-isoform of 41–43 kDa, though tobacco possesses only the β-isoform (Portis, 2003). The two isoforms differ by the presence of a carboxy-terminal extension containing redox-sensitive cysteine (Cys) residues in the former (Portis, 2003). Although both are capable of activating Rubisco (Zhang and Portis, 1999), expression of the isoforms often differs slightly. Previous studies in rice have shown that the expression level of mRNA encoding the α-isoform increases substantially after 24 h of heat treatment, whereas expression of the β-isoform declines (Scafaro et al., 2010) or is unaffected (Wang et al., 2010). This finding suggests differential regulation of the α-isoform gene, at least with regard to heat treatment. The soybean isoforms of RCA, *GmRCA*α, and *GmRCA*β, are encoded by separate genes, and bioinformatics analysis of their promoters has revealed a heat shock element in the former that is not present in the latter (Chao et al., 2014). In the present study, the expression pattern of the *GmRCA*α promoter was analyzed in soybean and *Arabidopsis*. While investigating the network regulating *GmRCA*α expression, we identified two TFs, GmbZIP04g and GmbZIP07g, that bind to the promoter region. These results might help us better understand the complicated regulatory network of the soybean RCA gene, *GmRCA*α.

## Materials and Methods

### Plant Material and Plant Growth Conditions

Soybean cultivar Kefeng No. 1 was used in all experiments in this study, including promoter analysis, gene cloning and gene expression analysis. The seeds of this cultivar were provided by Soybean Research Institute, Nanjing Agricultural University, China, and grown under natural conditions in the field at Jiangpu Experimental Station, Nanjing Agricultural University. At the R2 stage, the mature upper third of leaves were sampled at different times (0:00, 6:00, 12:00, and 18:00) to determine *GmbZIP04g, GmbZIP07g*, and *GmRCA*α gene expression levels during a single day.

*Arabidopsis thaliana* (Col-0) was used as the wild type. Sterilized seeds were incubated on MS medium at 4°C for 3 days before being transferred to a growth chamber (25°C/22°C, 300 μmol photons m^-2^ s^-1^, 70% relative air humidity and 12-h/12-h light/dark).

### Hormone and Abiotic Treatments

Soybean seeds were germinated in plugs containing a mixture of peat and vermiculite (3:1, v/v), and once the cotyledons were fully expanded, the soybean seedlings were selected and pre-cultured in one-half-strength Hoagland’s nutrient solution for 3 days. The uniformly grown seedlings were washed and transferred to different abiotic conditions. PEG treatment was carried out by transferring seedlings to water supplemented with 15% PEG, and leaves were sampled after 0.5 and 2 h. For ABA and high-salt treatments, seedlings were transferred to water supplemented with 250 mM NaCl and 100 μM ABA solutions, respectively. Control seedlings were submerged in water, and leaves were sampled at 0, 0.5, 2, 3, and 6 h. All samples were frozen in liquid nitrogen and stored at -80°C until further use for RNA extraction.

### RNA Preparation and RT-PCR

Total RNA was isolated from soybean leaf with an RNA Plant Extraction Kit (Tiangen, China), and approximately 2 μg of purified total RNA was reverse transcribed using AMV reverse transcriptase (Takara) according to the supplier’s instructions. RT-PCR was performed as described previously (Yin et al., 2010). The primers used for RT-PCR are listed in Supplementary Table S1. The soybean endogenous reference gene *tubulin* (GenBank: AY907703.1) was used as a control, and three technical replicates were performed.

### Y1H Assay

A soybean cDNA library was constructed from RNAs of the mature upper-third leaves at the full-seed stage (R6 stage) using CloneMiner II cDNA Library Construction Kit (Invitrogen). The cDNA library was then cloned into pGADT7-Rec2-DEST to generate the library for Y1H screening.

DNA fragments of the *GmRCA*α promoter and the pG-box and pmG-box were cloned into the pAbAi vector, then linearized by BstBI digestion, and transformed into Y1HGold to generate reporter strains. The pGmRCAα strain was then transformed with the library. For the Y1H assay, positive colonies were selected on yeast Leu dropout medium supplemented with 250 ng/mL AbA (Clontech). To reconfirm the Y1H screening results, the full-length coding sequence of putative candidates (GmbZIP04g and GmbZIP07g) was cloned into pGADT7, and Y1H assays were performed according to the manufacturer’s instructions.

### Plasmid Construction and Plant Transformation

To generate constructs to examine the expression patterns of *GmRCA*α, PCR was performed using KOD polymerase (Toyobo, Japan) and the primers listed in Supplementary Table S1, the amplifications were then digested with BamHI/PstI. As a reporter, the fragments were fused with the *GUS* gene in the binary vector pCAMBIA1381z (Cambia, Australia); four constructs, *GmRCA*α*_pro_(–2205)::GUS, GmRCA*α*_pro_(–889)::GUS, GmRCA*α*_pro_(–157)::GUS* and pCAMBIA1381z were transformed into *Agrobacterium tumefaciens* strain EHA105 using the freeze-thaw method and then transformed into *Arabidopsis* using the floral dip method (Clough and Bent, 1998). T1 seeds were collected and selected on MS medium containing 25 mg/mL hygromycin (hyg) and then tested by amplification of a 457-bp fragment using primer pairs targeting the promoter and GUS sequences. T3 and T4 homozygous lines were used for further study. The *GmRCA*α*_pro_(–2205)::GUS* construct was transformed into soybean cotyledonary nodes, and the transformation was performed following a previously described method (Zhang et al., 1999).

### Promoter Transactivation Assays Using *Arabidopsis* Mesophyll Protoplasts

To generate firefly luciferase reporter construct, a 2205-bp sequence upstream of *GmRCA*α was PCR amplified using the primers listed in Supplementary Table S1, digested with BamHI and NcoI, and then cloned into pRD29A-*LUC* (EF090409) to replace the RD29A promoter in the vector. To prepare effector TFs, the entire coding region of *GmbZIP04g* and *GmbZIP07g*, without the stop codon TAG, were amplified by PCR. Both of the fragments were digested with BamHI and ligated to the same digested HBT95::sGFP(S65T)-NOS vector. The pPTRL (*Renilla reniformis* Luciferase driven by 35S promoter) was used as internal control (Fang et al., 2015).

*Arabidopsis* protoplasts were isolated and transformed according to the method described by Wang et al. (2015). The effector, reporter and internal control were co-transformed into *Arabidopsis* protoplasts with 10, 5, and 0.5 μg, respectively. The protoplasts were incubated under weak light for 12–16 h before harvesting. Dual luciferase assay was performed with a Dual-luciferase assay system kit (Promega) according to the manufacturer’s instructions. Briefly, the transformed protoplasts were centrifuged at 12000 × *g* for 15 s at room temperature, and the supernatant was removed. Then add 100 μl of the Passive Lysis Buffer with further homogenization. Twenty microliters of the lysate was mixed with 100 μl of LAR II, and the firefly luciferase (LUC) activity was measured using a GloMax 20/20 luminometer (Promega). Then 100 μl of Stop & Glo Reagent was added to the reaction, and the *Renilla* luciferase (REN) activity was measured. Four repeats were performed. Differences were analyzed by one-way ANOVA, with *post hoc* analysis using SPSS16.0. ^∗∗^ denotes significant difference at *P* = 0.01.

### Subcellular Localization

The same effector TF constructs were used to investigate GmbZIP04g and GmbZIP07g localization. *Arabidopsis* protoplasts were isolated and transformed as promoter transactivation assays. The protoplasts were incubated under weak light for 12 to 16 h and observed with an LSM 780 Exciter confocal laser scanning microscope (Zeiss). The excitation wavelength used for GFP was 488 nm.

### Histochemical GUS Staining and Activity Assay

Transgenic *Arabidopsis* expressing GUS expression constructs were collected at several growth stages and subjected to GUS staining. GUS staining in both transgenic *Arabidopsis* and soybean cotyledonary nodes was performed as described previously, with some modification (Chao et al., 2014). Plant samples were soaked at 37°C for 12 h in the GUS assay solution which included 0.5 mg/ml 5-bromo-4-chloro-3-indolyl glucuronide, 0.1% Triton X-100, 1 mM K_3_[Fe(CN)_6_], 1 mM K_4_[Fe(CN)_6_], 10 mM EDTA and 50 mM sodium phosphate buffer (pH 7.0) in darkness. The staining solution was then replaced by 75% ethanol to remove chlorophyll. Ethanol washing was repeated 3–5 times for 6 h. Then GUS staining was observed with an Olympus SZX12 stereomicroscope and photographed with a digital camera (CoolSNAP, RS photometrics). For transgenic *Arabidopsis* plants, quantitative analysis of GUS activity was detected according to the method of Jefferson et al. (1987).

### Transactivation Activity Assay in Yeast

The transactivation activity assay was performed using the Matchmaker Gold Yeast Two-Hybrid System (Clontech, USA). To construct BD-GmbZIP04g and BD-GmbZIP07g, the full-length coding sequence of *GmbZIP04g* and *GmbZIP07g* was amplified by PCR and then fused into pGBKT7 vector, using the same primer pairs with that of pGADT7 constructs (Supplementary Table S1). Combinations of BD-GmbZIP04g and AD, BD-GmbZIP07g and AD, positive and negative control vectors were transformed into yeast strain AH109, respectively. The transformed yeasts were grown on SD/-Trp/-Leu/X-α-gal (DDO/X) and SD/-Trp/-Leu/-His/-Ade/X-α-gal (QDO/X) agar plates. After 3 days growth in dark, the plates were photographed.

## Results

### GUS Expression Driven by the *GmRCA*α Promoter in Soybean Cotyledonary Nodes

**FIGURE 1:**
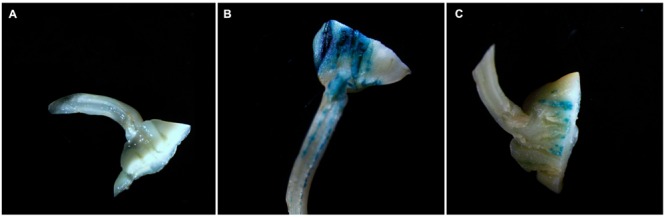
**Histochemical staining of soybean cotyledonary nodes driven by the promoter sequence of *GmRCA*α. (A)** Negative control pCAMBIA1381z; **(B)** positive control pCAMBIA1301; **(C)** *GmRCA*α*_pro_(–2205)::GUS*.

To determine whether the promoter sequence of the *GmRCA*α gene is able to activate GUS expression, we carried out a transient assay by cloning a 2205-bp promoter fragment of *GmRCA*α, referred to as *GmRCA*α*_pro_(–2205)::GUS*, upstream of the GUS reporter gene. Subsequent staining results indicated that *GmRCA*α*_pro_(–2205)::GUS* can drive GUS expression in soybean cotyledonary nodes (**Figure 1**).

### Expression Patterns and Deletion Analysis of the *GmRCA*α Promoter in *Arabidopsis*

**FIGURE 2:**
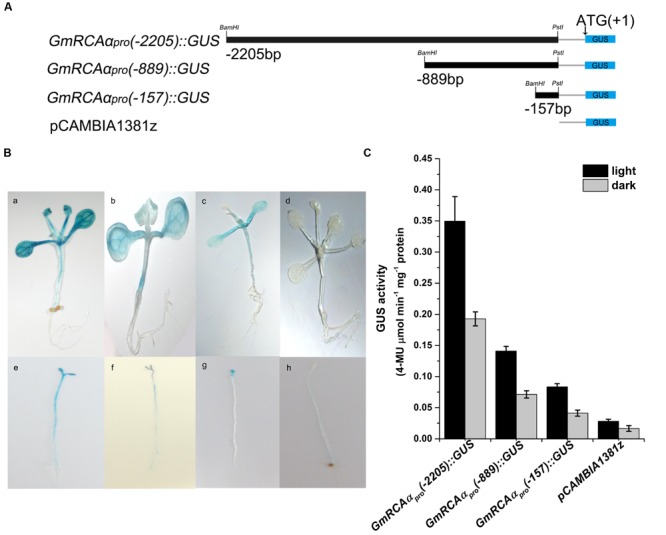
**GUS activity in transgenic *Arabidopsis*. (A)** The diagram shows *GmRCA*α promoter 5′ terminal deletion mutant constructs fused with the reporter gene *GUS*. **(B)** Histochemical staining of transgenic *Arabidopsis*. (a–d) Green seedlings grown under a 12-h/12-h light/dark photoperiod; (e–h) etiolated seedlings grown in the dark. a and e, b and f, c and g, and d and h represent transgenic seedlings expressing *GmRCA*α*_pro_(–2205)::GUS, GmRCA*α*_pro_(–889)::GUS, GmRCA*α*_pro_(–157)::GUS* and pCAMBIA1381z, respectively. **(C)** GUS activity assay of the green and etiolated seedlings indicated in **(B)**. Error bars represent SE (*n* = 3). 4-MU, 4-methylumbelliferone.

To analyze the function of the *GmRCA*α promoter, two deletion mutants were fused with the GUS reporter gene [named *GmRCA*α*_pro_(–889)::GUS* and *GmRCA*α*_pro_(–157)::GUS*] (**Figure 2A**) and transformed into *Arabidopsis*, as was *GmRCA*α*_pro_(–2205)::GUS*.

**FIGURE 3:**
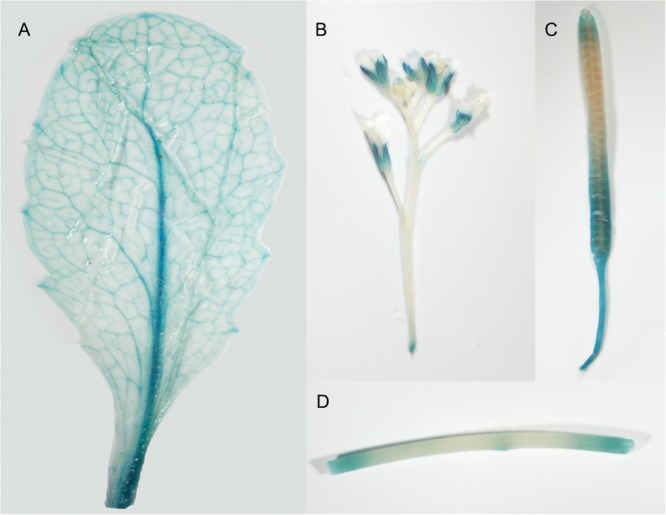
**GUS staining for *GmRCA*α*_pro_(–2205)::GUS* activity in transgenic *Arabidopsis*. (A)** A mature leaf from a 2-week-old seedling; **(B)** flowers; **(C)** mature silique; **(D)** stem.

β-Glucuronidase staining revealed a detailed temporal and spatial expression pattern for *GmRCA*α*_pro_(–2205) ::GUS*. In young seedlings, staining was observed mainly in green tissues, including cotyledons, true leaves and hypocotyls, but not in roots (**Figure 2B**), whereas in mature plants, GUS activity was found in leaves, particularly in vascular tissues, stems, green siliques and flower sepals (**Figure 3**).

β-Glucuronidase staining of 7-day-old green seedlings grown under a 12-h/12-h light/dark photoperiod showed that *GmRCA*α*_pro_(–2205)::GUS, GmRCA*α*_pro_(–889)::GUS* and *GmRCA*α*_pro_(–157)::GUS* were all able to drive GUS expression (**Figure 2B**), with expression driven by *GmRCA*α*_pro_(–2205)::GUS* being stronger than that by *GmRCA*α*_pro_(–889)::GUS* and staining for *GmRCA*α*_pro_(–889)::GUS* being stronger than that for *GmRCA*α*_pro_(–157)::GUS* (**Figure 2C**). Etiolated dark-grown seedlings showed similar staining. These results indicated that positive regulatory elements may be located in the regions extending from -2205 to -889 and from -889 to -157.

### Identification of bZIP TFs Interacting with the *GmRCA*α Promoter

To identify putative TFs regulating *GmRCA*α expression, a Y1H assay was carried out to identify proteins interacting with the *GmRCA*α promoter. A 2205-bp fragment upstream the ATG start codon of *GmRCA*α was initially used as bait, but 500 ng/mL AbA did not suppress basal expression in the reporter strain. Then, a 1459-bp *GmRCA*α promoter was isolated and applied for screening a soybean cDNA library. Among the positive clones (Supplementary Table S2), four TFs belonging to the basic leucine zipper (bZIP) family appeared 17 times during screening and were thus analyzed further. The corresponding gene names are Glyma04g04170, Glyma06g04353 (*GmbZIP71*) (Liao et al., 2008), Glyma07g33600, and Glyma02g14880 (*GmbZIP1*) (Gao et al., 2011), which we refer to herein as *GmbZIP04g, GmbZIP71, GmbZIP07g*, and *GmbZIP1*.

**FIGURE 4:**
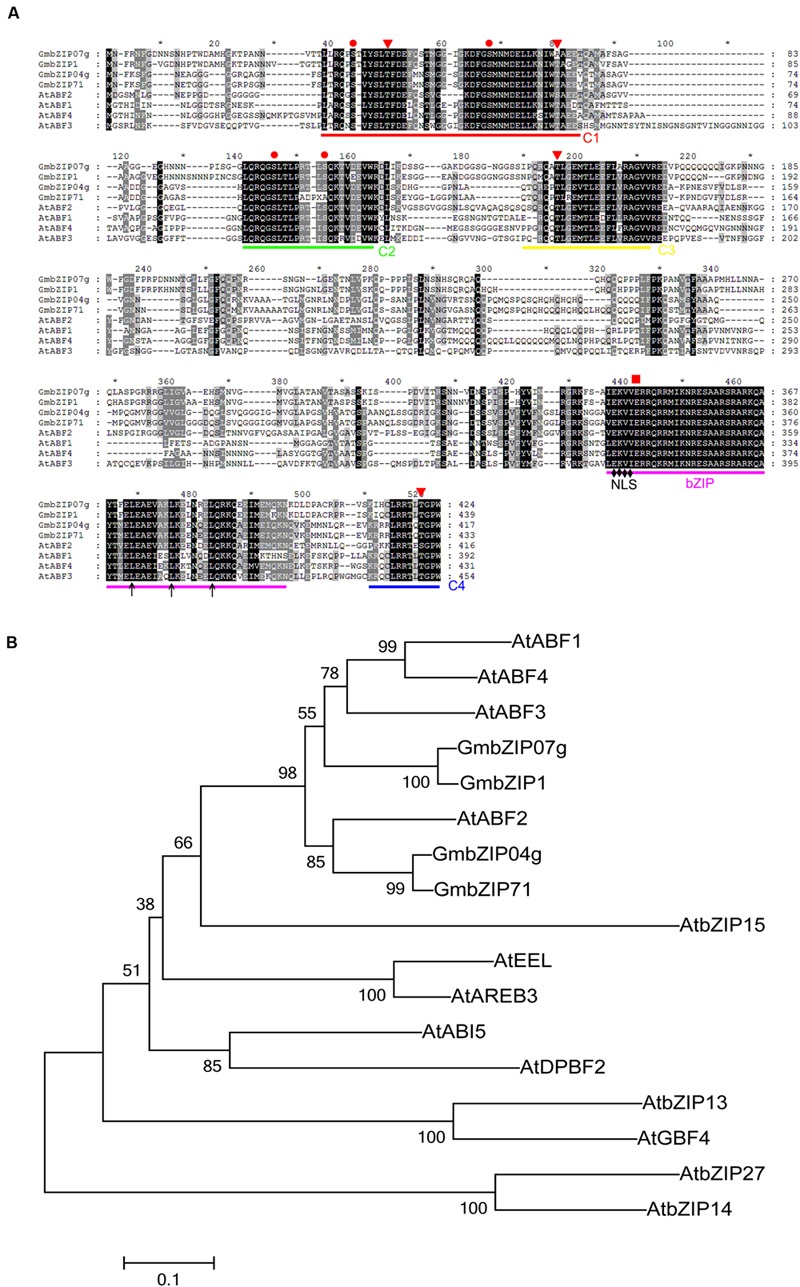
**Amino acid sequence alignment and phylogenetic analysis of GmABFs. (A)** Sequence alignment of GmABFs and AtABFs. The alignment was generated using CLUSTALX (version 1.83) and viewed using the GeneDOC program (version 2.6.0.2). The basic region and the three heptad leucine repeats, three important bZIP signatures, are shown as purple line and arrows, respectively. C1, C2, C3, and C4 are conserved regions containing serine (●) and threonine (▼) residues. The NLS, the putative nuclear localization signal, is indicated by a black diamond. The red square indicates the boundary between the N- and C-terminal of GmbZIP07g. ^∗^ indicates that this position is an odd multiple of 10. **(B)** Phylogenetic tree of GmABFs and *Arabidopsis thaliana* bZIP group A proteins using MEGA6.06. The protein sequences were obtained from Phytozome (http://www.phytozome.net/) and The Arabidopsis Information Resource (http://www.arabidopsis.org/).

Multiple alignments for GmbZIP04g, GmbZIP71, GmbZIP07g, GmbZIP1, and four other ABF proteins of *Arabidopsis thaliana* revealed high sequence identity. As shown in **Figure 4A**, these four proteins possess a conserved bZIP domain at the C-terminus, a putative nuclear localization signal (NLS) and four additional conserved domains, three at the N-terminal half (C1, C2, and C3) and one at the C-terminal end (C4) (Fujita et al., 2005; Huang et al., 2010; Gao et al., 2011). Based on the characteristics of the conserved domains, we assigned these four bZIP proteins of soybean to the ABF subgroup of the bZIP family. A phylogenetic tree constructed using amino acid sequences of GmABFs (GmbZIP04g, GmbZIP71, GmbZIP07g, and GmbZIP1) and bZIP groupA proteins from *Arabidopsis thaliana* (Jakoby et al., 2002) presented that GmABFs are most closely related to AtABF1-4 proteins (**Figure 4B**), with GmbZIP1 having high similarity to GmbZIP07g (86.00%) and GmbZIP71 sharing 87.76% similarity with GmbZIP04g.

**FIGURE 5:**
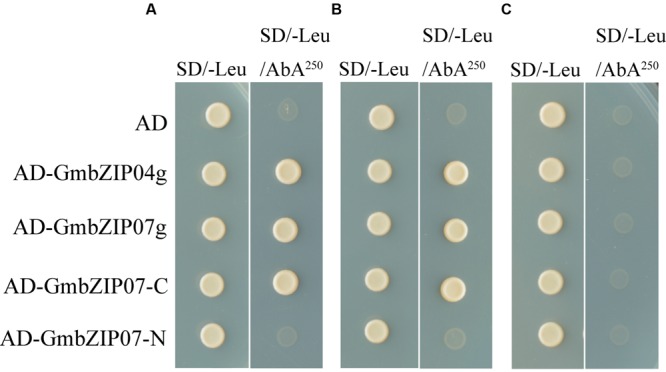
**Identification of proteins interacting with pGmRCAα, pG-box, and pmG-box reporter strains by a Y1H assay.** The promoter of GmRCAα, G-box sequence, and mutated G-box were cloned upstream of the AbA resistance (AbA^r^) gene and integrated into Y1HGold to generate reporter strains. The reporter strains were transformed with the effector transcription factors (TFs) indicated on the left, and growth was recorded for 3 days at 30°C in the absence or presence of 250 ng/mL AbA. **(A)** pGmRCAα reporter strain. **(B)** pG-box reporter strain. **(C)** pmG-box reporter strain.

Based on the phylogenetic relationship, we verified whether two of these bZIP TFs, GmbZIP04g, and GmbZIP07g, are able to bind the *GmRCA*α promoter using the full-length ORF in a second Y1H assay (**Figure 5A**). Y1H assays were also conducted to identify which TF region is responsible for binding to the *GmRCA*α promoter. Given the sequence similarity (44.42%) between GmbZIP04g and GmbZIP07g and their highly conserved domains, GmbZIP07g was selected for further analyses. Two fragments of GmbZIP07g were generated based on the bZIP domain of bZIP family proteins; the corresponding constructs were named GmbZIP07-N(1-345AA) and GmbZIP07-C(346-430AA). Deletion of the C-terminal 84 amino acids of GmbZIP07g resulted in a lack of binding to the *GmRCA*α promoter (**Figure 5A**). Thus, GmbZIP07g possesses binding activity, and this activity resides within the C-terminal region.

### GmbZIP04g and GmbZIP07g Interact with a Specific Sequence in the *GmRCA*α Promoter

Numerous bZIP proteins have been reported as interacting with the ABRE or G-box (CACGTG) (Huang et al., 2010; Dai et al., 2013). According to a bioinformatics analysis of the *GmRCA*α promoter, only one conserved ABRE/G-box (CACGTG) is present in the 1459-bp fragment. Therefore, we examined whether GmbZIP04g and GmbZIP07g bind to a 21-nt G-box/ABRE-containing sequence.

Both this G-box containing sequence (pG-box, AGTTGC CACGTGGCAGCCAAG, G-box core sequence was underlined) and a mutated G-box sequence (pmG-box, AGTTGC AACGACGCAGCCAAG, mutant sequence of the G-box is underlined) were prepared as reporter strains, and DNA-binding activity was determined using a Y1H assay. **Figures 5B,C** shows that the clones harboring GmbZIP04g, GmbZIP07g, and GmbZIP07-C grew well on SD/-Leu/AbA^250^ medium with the pG-box reporter strain, whereas GmbZIP07-N and the negative control (empty pGADT7 AD vector) did not grow. Thus, GmbZIP04g and GmbZIP07g specifically bound the G-box/ABRE-containing sequence through the C-terminus of GmbZIP07g. Furthermore, no clones grew with the pmG-box reporter strain, indicating that the CACGTG motif is essential for DNA binding.

### GmbZIP04g and GmbZIP07g Are Localized in the Nucleus and Exhibit Transactivation Activity in Yeast

**FIGURE 6:**
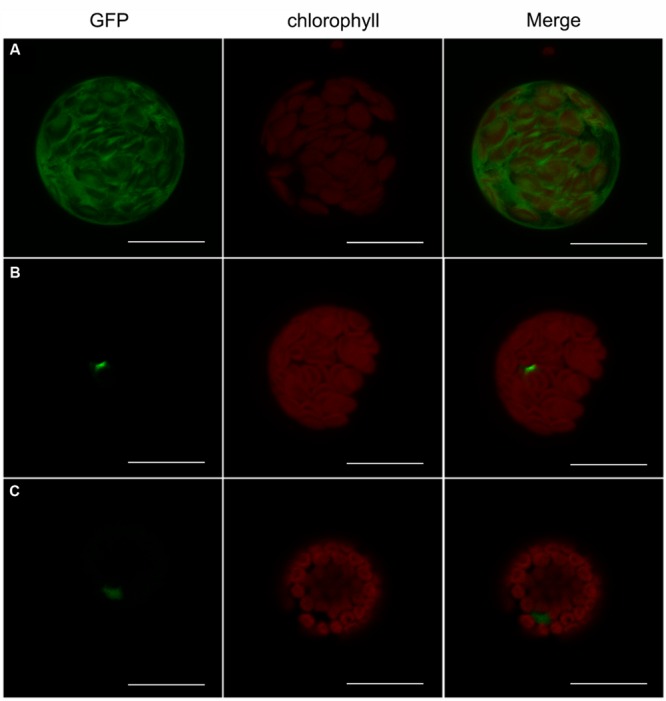
**Nuclear localization of GmbZIP04g and GmbZIP07g in *Arabidopsis* leaf protoplasts.** Confocal laser scanning microscope images of leaf protoplasts transiently expressing **(A)** HBT95::sGFP(S65T)-NOS, **(B)** GmbZIP04g-sGFP, and **(C)** GmbZIP07g-sGFP. Column 1, GPF signal (green); column 2, chlorophyll autofluorescence (red); column 3, merged GFP and chlorophyll signals. Scale bars represent 20 μm.

GmbZIP04g and GmbZIP07g are TFs, and both have a nuclear localization signal near the C terminus, suggesting that they may be localized to the nucleus. To determine the intracellular localization of GmbZIP04g and GmbZIP07g, we transiently transformed GmbZIP04g-sGFP and GmbZIP07g-sGFP into *Arabidopsis* protoplasts and found GmbZIP04g and GmbZIP07g in the nucleus (**Figure 6**).

**FIGURE 7:**
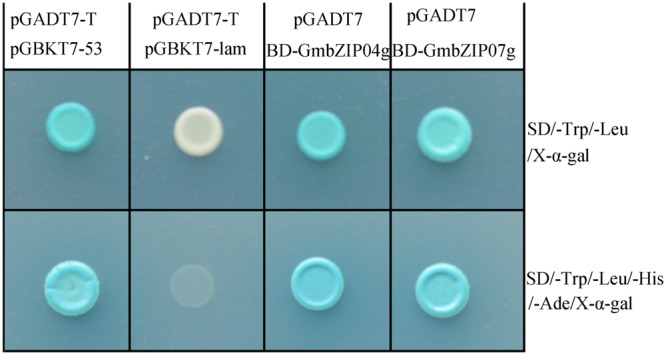
**Transactivation assay of GmbZIP04g and GmbZIP07g in the yeast strain AH109.** Positive control (pGADT7-T and pGBKT7-53); Negative control (pGADT7-T and pGBKT7-lam); BD-GmbZIP04g and pGADT7; BD-GmbZIP07g and pGADT7. The SD/-Trp/-Leu/X-α-Gal (DDO/X, Upper) and SD/-Trp/-Leu/-His/-Ade/X-α-Gal (QDO/X, Lower) plates were incubated at 30°C for 3 days and then visualized.

To evaluate whether GmbZIP04g and GmbZIP07g are able to activate transcription, full-length GmbZIP04g and GmbZIP07g were fused to the GAL4 DNA-binding domain (GAL4-BD) in the pGBKT7 vector. The constructs were co-transformed into yeast strain AH109 with pGADT7, then screened on DDO/X and QDO/X media. By X-α-Gal assay, the GmbZIP04g and GmbZIP07g yeast clones were weak blue in colour (**Figure 7**), indicating that GmbZIP04g and GmbZIP07g possess transactivation activity in yeast.

### Expression Patterns of *GmbZIP04g, GmbZIP07g*, and *GmRCA*α

**FIGURE 8:**
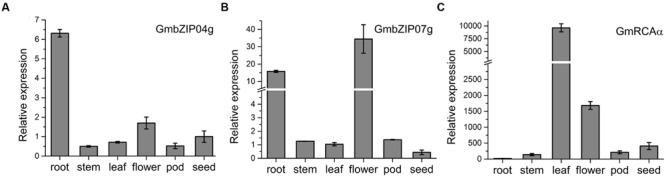
**Tissue-specific expression of *GmbZIP04g***(A)**, *GmbZIP07g***(B)**, and *GmRCA*α **(C)**.** Transcript expression levels were measured by RT-PCR. Total RNA was isolated from soybean roots, stems and leaves harvested at the seedling stage, from flowers harvested at the R2 stage (flowering) and from seeds harvested at the R6 stage (full seed). *Tubulin* was used as an internal control, and error bars represent the standard error of three independent repetitions.

**FIGURE 9:**
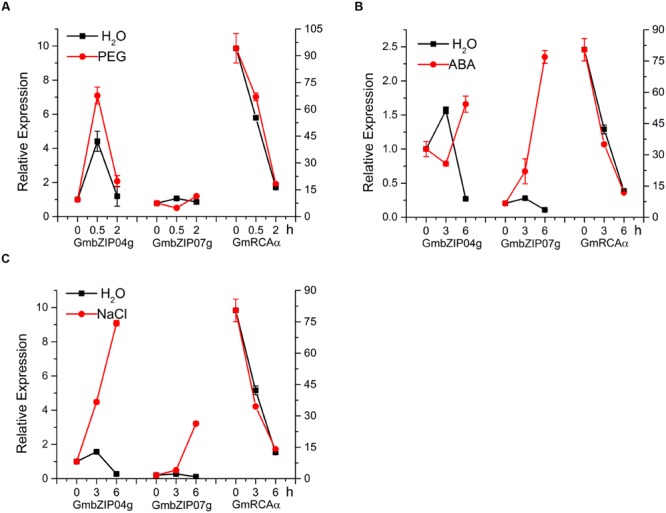
**Time-course expression analysis of *GmbZIP04g, GmbZIP07g*, and *GmRCA*α. (A–C)** Soybean seedlings were exposed to PEG (15%), NaCl (250 mM), ABA (100 μM), and water treatments. *GmbZIP04g* expression under water treatment at 0 h was used as the reference for the basal expression level. The left *y*-axis indicates the relative expression level of *GmbZIP04g and GmbZIP07g*, and the right *y*-axis represents the expression of *GmRCA*α. *Tubulin* was used as an internal control. Error bars represent the SE (*n* = 3).

**FIGURE 10:**
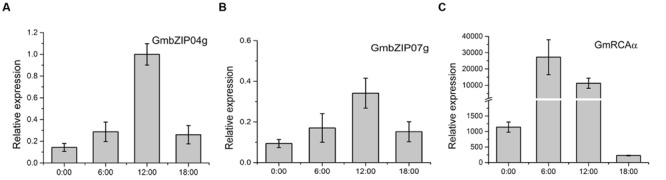
**Diurnal pattern of *GmbZIP04g***(A)**, *GmbZIP07g***(B)**, and *GmRCA*α **(C)** mRNA accumulation in soybean leaves.** Total RNA was extracted at 0:00, 6:00, 12:00, and 18:00 from the upper-third leaves of soybean plants at the R2 stage. *Tubulin* was used as an internal control, and error bars represent the SE (*n* = 3).

Tissue analysis of *GmbZIP04g* and *GmbZIP07g* revealed expression in root, stem, flower, leaf, and seed (**Figures 8A,B**); as *GmRCA*α was mainly expressed in leaf (**Figure 8C**), we chose this tissue for further study. The expression of *GmbZIP04g, GmbZIP07g*, and *GmRCA*α was analyzed in plants subjected to PEG, ABA, and NaCl stresses. As shown in **Figures 9A–C**, *GmRCA*α exhibited a slight change at 3 h and was almost the same as the control at 6 h under all these treatments. After PEG treatment, the expression of *GmbZIP04g* was higher than that in the control, and *GmbZIP07g* showed a lower expression at 0.5 h followed a higher level at 2 h compared to the control. The transcript level of *GmbZIP04g* was lower than that in the control at 3 h but was significantly higher at 6 h when subjected to ABA treatment. With ABA and NaCl treatments, continuous accumulation of *GmbZIP07g* transcript was observed at 3 and 6 h, and a similar pattern for *GmbZIP04g* was observed in response to NaCl treatment. The upper-third leaves of soybean were sampled at 6-h intervals for 24 h, and RT-PCR assays were then performed. The results showed that the transcripts of *GmbZIP04g* and *GmbZIP07g* increased from 0:00 to 12:00, reaching a peak at noon and then decreasing, whereas the maximal level of *GmRCA*α was detected at 6:00 (**Figures 10A–C**). Our results suggest that *GmbZIP04g, GmbZIP07g*, and *GmRCA*α may be regulated in response to abiotic stresses and light.

## Discussion

The genes encoding two RCA isoforms, *GmRCA*α and *GmRCAβ*, have been previously characterized (Yin et al., 2010). In addition, the levels of *GmRCA*α and *GmRCAβ* gene expression have been positively correlated with Rubisco initial activity, P_N_, chlorophyll fluorescence parameters and seed yield, indicating that RCA genes may play important roles in regulating soybean photosynthetic capacity and enhancing soybean productivity (Yin et al., 2010; Chao et al., 2014). Here, we report the expression patterns of *GmRCA*α by examining the activity of its promoter. The *GmRCA*α promoter was able to drive GUS expression in soybean cotyledonary nodes (**Figure 1**), and GUS staining in *Arabidopsis* revealed expression mainly in green tissues (**Figure 2**), which is similar to that of *Arabidopsis* and rice *RCA* genes (Liu et al., 1996; Yang et al., 2012). We also found that a 157-bp fragment upstream of the ATG to be sufficient for tissue-specific expression in transgenic *Arabidopsis*. Minimal promoters conferring *RCA* gene expression in *Arabidopsis*, rice, and spinach were previously identified within 317 bp, 297 bp, and 294 bp upstream of the transcription initiation site (Orozco and Ogren, 1993; Liu et al., 1996; Yang et al., 2012). These results suggest that basal and/or important elements are located proximal to the 5′-untranslated region in both dicots and monocots.

Although numerous studies have shown that RCA gene and/or protein expression is affected by various factors, such as biotic (Mitra and Baldwin, 2014) and abiotic (Ji et al., 2012; Chen et al., 2015) stresses, there are no reports to date on the direct transcriptional regulation of *RCA*. Regulatory TFs activate or repress transcription of their target genes by binding to *cis*-elements, which are frequently located in a gene’s promoter. In this study, Y1H screening was performed to investigate the upstream regulators of *GmRCA*α. We isolated and identified four bZIP proteins, GmbZIP1, GmbZIP04g, GmbZIP71, and GmbZIP07g, as interacting with the promoter sequence of *GmRCA*α (**Figure 5**). To the best of our knowledge, this is the first report of interaction between the *RCA* promoter and a TF. Furthermore, we found that it is the C-terminal conserved bZIP domain of GmbZIP07g, but not the N-terminus, that interacts with the *GmRCA*α promoter (**Figure 5**). This result suggests that the C-terminal of GmbZIP07g may play a crucial role in downstream promoter elements binding.

Expression QTL analysis can provide tremendous insight into the biology of gene regulation, indeed, measurements of gene expression of a population helps us to learn which types of SNPs are most likely to affect gene regulation (Gilad et al., 2008). Biological processes are complex networks of physical interactions between various molecules, and one type of interaction occurs between TFs and their target DNA sites, generally *cis*-elements in the promoter of a gene, to up-regulate or down-regulate expression. The Y1H assay used in this study is one method for detecting protein–DNA interaction (Reece-Hoyes and Marian Walhout, 2012). However, GmbZIP04g, GmbZIP07g, GmbZIP1, and GmbZIP71 were not detected by eQTLs in our previous study (Yin et al., 2010; Chao et al., 2014); possible explanations for this might be the lower marker density (Zhao et al., 2011), a statistical reason that many distal eQTLs are missed (Gilad et al., 2008), and non-genetic factors such as environmental variation.

Plant bZIP family members regulate various processes, such as pathogen defense, light and abiotic stress signaling, hormone signaling, energy metabolism, and development, including flowering and seedling maturation (Jakoby et al., 2002; Llorca et al., 2014). Among light-regulated bZIP proteins in *Arabidopsis* (Jakoby et al., 2002) and rice (Nijhawan et al., 2008), *AtHY5* has been well documented as a critical regulator of photomorphogenesis (Chen et al., 2008). The conserved sequence motif CACGTG, generally known as the G-box or “ABA-responsive” element (ABRE), is present in the promoter regions of many light-regulated genes (Choi et al., 2000; Uno et al., 2000; Jiao et al., 2007; Xu et al., 2014). Numerous bZIP class proteins have been isolated, and experiments have shown that these factors can interact with the ABRE or G-box both *in vivo* and *in vitro* (Huang et al., 2010; Dai et al., 2013). Previous studies have reported that many bZIP proteins bind to a G-box/ABRE element, which contains an ACGT core motif. For example, AtABF proteins 1–4 were isolated by a Y1H system using a prototypical ABRE, the Em1a element (GGACACGTGGCG) (Choi et al., 2000). In the present study, we also found that GmbZIP04g, GmbZIP07g and the C-terminus of GmbZIP07g interact with a 21-nt DNA sequence containing the G-box/ABRE element (**Figure 5**) using a Y1H assay, suggesting that GmbZIP04g and GmbZIP07g might directly regulate the expression of *GmRCA*α through this region. The bZIP TFs GmbZIP04g, and GmbZIP07g are localized in the nucleus and exhibit transactivation activity in yeast cells (**Figures 6** and **7**), in agreement with their homologs, such as AtABF2 and AtABF4 (Uno et al., 2000; Yoshida et al., 2010).

All of *GmRCA*α, *GmbZIP04g*, and *GmbZIP07g* are expressed in leaf (**Figures 8A–C**), suggesting a functional role in this tissue. *GmbZIP04g* and *GmbZIP07g* revealed a higher expression in root, while the expression of *GmRCA*α in root was almost undetectable, we suppose that GmbZIP04g and GmbZIP07g may have other interacting promoters in addition to that of *GmRCA*α. And a possible explanation for low expression of *GmRCA*α in root may be due to the function of its unknown repressors. As the ABRE is involved in abiotic stresses (Choi et al., 2000; Gao et al., 2011), it would be interesting to investigate the involvement of GmbZIP04g, GmbZIP07g, and GmRCAα in abiotic stresses in soybean. The levels of *GmbZIP04g, GmbZIP07g*, and *GmRCA*α transcripts were examined in soybean seedlings upon abiotic stresses, and RT-PCR analysis showed that expression of *GmbZIP04g* and *GmbZIP07g* was highly induced after ABA and NaCl treatments, whereas the transcriptional levels of *GmRCA*α appeared to be unchanged (**Figure 9**). *GmRCA*α mRNA expression only showed a slight change after PEG and ABA treatments. One possible explanation for the lack of an immediate effect on the transcriptional levels of *GmRCA*α with *GmbZIP04g* and *GmbZIP07g* induction may due to competition with other TFs that activate or repress the expression of *GmRCA*α. In addition, ABFs are extensively regulated at the post-transcriptional level (Fujita et al., 2013), and as a result, an increase in transcript levels may not always correlate directly with an increase in protein levels. Furthermore, ABFs or AREBs in *Arabidopsis* were shown to interact with other proteins, form homo- and/or heterodimers (Kim et al., 2004a; Choi et al., 2005; Lee et al., 2010; Yoshida et al., 2010), suggesting a potential mechanism for generating diversity in these protein regulation networks. Such interaction between the two GmbZIP proteins may be examined in the future. As RCA diurnally regulated in higher plants (Chao et al., 2014; Yin et al., 2014), we examined the expression levels of *GmbZIP04g* and *GmbZIP07g* at different time points. Our RT-PCR analysis showed that *GmbZIP04g* and *GmbZIP07g* expression increased from midnight to noon, suggesting that these genes may have a diurnal expression pattern similar to that of *GmRCA*α, ABF3 (Seung et al., 2012) and *GmbZIP1* (Marcolino-Gomes et al., 2014). However, the maximal transcript level of *GmRCA*α appeared at 6:00, earlier than *GmbZIP04g* and *GmbZIP07g*, both of which reached their peaks at 12:00; thus, we infer that other TFs may be involved in this process.

In higher plants, *Arabidopsis* protoplasts system was widely used to study the regulation between TFs and its binding promoter (Yi et al., 2010; Figueiredo et al., 2012; Wang et al., 2015). A transient assay in *Arabidopsis* protoplasts was performed to investigate the effects of GmbZIP04g and GmbZIP07g on *GmRCA*α promoter activity, and the LUC/REN values of GmbZIP04g and GmbZIP07g were higher than that of the empty vector (Supplementary Figure S1 and Supplementary Table S3). Further studies with transgenic approaches will help to understand the precise functions of GmbZIP04g and GmbZIP07g on the expression of *GmRCA*α.

In recent years, cross-talk between light and ABA as well as sugar and ABA are being increasingly studied. The plant hormone ABA plays essential roles during many phases of the life cycle and adaptation to environmental stresses, such as stomatal closure in response to drought. It was reported that light and ABA signaling are integrated at the promoters of *HY5* (Chen et al., 2008) and *ABI5* (Xu et al., 2014), which are bZIP proteins, during seed germination and early seedling development. Carbohydrates are the end products of photosynthesis, and ABF proteins are also involved in sugar signaling (Kim et al., 2004b). In addition, ABA and sugar have been shown to suppress many photosynthetic genes, including *RBCS* and *CAB* (Rook et al., 2006), and bZIP TFs were found to mediate the effects of sugar signaling on gene expression and metabolite content (Hanson and Smeekens, 2009). Moreover, interaction between ABA and Rubisco was recently identified biochemically (Galka et al., 2015), and RCA is a type of chaperone that functions to promote and maintain the catalytic activity of Rubisco, which is a crucial photosynthesis protein (Portis, 2003). Our research identified two bZIP TFs, GmbZIP04g and GmbZIP07g, interacting with the soybean *GmRCA*α promoter in yeast. However, it remains largely unknown how these two TFs are involved in the regulation of *GmRCA*α gene expression in soybean. Further studies on the relationship between GmRCAα and the TFs, GmbZIP04g and GmbZIP07g, in soybean are necessary to fully elucidate the complex regulatory mechanism of GmRCAα expression and to help unravel the function of these proteins in the regulation of photosynthesis processes.

## Author Contributions

DY, JZ, and MC conceived and designed the experiments. JZ, HD, HY, and YL performed the experiments. JZ, FH, HD, MC, and ZY wrote the paper. All authors read and approved the manuscript.

## Conflict of Interest Statement

The authors declare that the research was conducted in the absence of any commercial or financial relationships that could be construed as a potential conflict of interest.

## Abbreviations

4-MU: 4-methylumbelliferone
ABA: abscisic acid
AbA: aureobasidin A
GFP: green fluorescent protein
GUS: β-glucuronidase
RT-PCR: real-time PCR
