# Supplementary material for: Identification of Two bZIP Transcription Factors Interacting with the Promoter of Soybean Rubisco Activase Gene (GmRCAα)
Source: Front Plant Sci. 2016 May 17;7:628. doi: 10.3389/fpls.2016.00628 (PMC4868853; doi:10.3389/fpls.2016.00628)
Supplement: Supplementary file 1 [file Data_Sheet_1.DOCX]

**Identification of two bZIP transcription factors interacting with the promoter of soybean Rubisco activase gene (*GmRCAα*)**

Jinyu Zhang^1^, Hongyang Du^1^, Maoni Chao^2^, Zhitong Yin^3^, Hui Yang^1^, Yakai Li^1^, Fang Huang^1*^, Deyue Yu^1*^

***Correspondence:**

Deyue Yu

E-mail: dyyu@njau.edu.cn

Fang Huang

E-mail: fhuang@njau.edu.cn

**Supplemental Tables**

**Supplemental Table S1** Primer pairs used in this research. Red letters represent enzyme sites, and mutant G-box nucleotides were underlined.

|  | **Oligo names** | **sequences(5'->3')** | **enzyme site** |
| --- | --- | --- | --- |
| **promoter activity** | pGmRCAα-F  (1-2205) | 5' CGCGGATCCCACTTTAACGCATCTTTGGATCT 3' | BamHI |
|  | pGmRCAα-F  (1-889) | 5' CGCGGATCCATAGCAGGTAATTTGGAAGGACTAG 3' | BamHI |
|  | pGmRCAα-F  (1-157) | 5' CGCGGATCCGTGTGGATGTTAAGGATTCTACAACT3' | BamHI |
|  | pGmRCAα-R | 5' AAAACTGCAGGGCAGTGTAGTCCTACTCAGAGAGT3' | PstI |
|  | pTEST-F | 5' ACTCTCTGAGTAGGACTACACTGCC 3' | no |
|  | pTEST-R | 5' ATTTCACGGGTTGGGGTTTC 3' | no |
| **Y1H** | Y1H-F | 5' CCGGAGCTCAATATGCATGGTGGAGTGAAGG 3' | SacI |
| **reporter** | Y1H-R | 5' CCGCTCGAGGGCAGTGTAGTCCTACTCAGAGAGT 3' | XhoI |
| **strain** | ABRE-SF | 5' CAGTTGCCACGTGGCAGCCAAGC 3' | SacI |
|  | ABRE-XR | 5' TCGAGCTTGGCTGCCACGTGGCAACTGAGCT 3' | XhoI |
|  | mABRE-SF | 5' CAGTTGCAACGACGCAGCCAAGC 3' | SacI |
|  | mABRE-XR | 5' TCGAGCTTGGCTGCGTCGTTGCAACTGAGCT 3' | XhoI |
| **Transcriptional** | AD-GmbZIP04-F | 5' CCGGAATTCATGAATTTCAAGAGCTTTGGAA 3' | EcoRI |
| **factor** | AD-GmbZIP04-R | 5' CGCGGATCCCTACCACGGACCAGTTTGTGT 3' | BamHI |
|  | AD-GmbZIP07g-F | 5' CCGGAATTCATGAACTTCAGGAACTTTGGT 3' | EcoRI |
|  | AD-GmbZIP07g-R | 5' CGCGGATCCCTACCATGGTCCAGTAAGTGTC 3' | BamHI |
|  | AD-GmbZIP07g-2F | 5' CCGGAATTCAGGAGACAAAGGAGAATGAT 3' | EcoRI |
|  | AD-GmbZIP07g-1R | 5' CCGCTCGAGCTCAACCACTTTCTCTATGGC 3' | XhoI |
| **LUC** | pGmRCAα-BamHI-F | 5' GCGCGGATCCCACTTTAACGCATCTTTGGATCTGTTC 3' | BamHI |
|  | pGmRCAα-NcoI-R | 5' CGCGCCATGGGGCAGTGTAGTCCTACTCAGAG 3' | NcoI |
| **35S-GFP** | GmbZIP04g-HBT-F | 5' CGCGGATCCATGAATTTCAAGAGCTTTGGA 3' | BamHI |
|  | GmbZIP04gHBT-R | 5' CGCGGATCCCCACGGACCAGTTTGTGTT 3' | BamHI |
|  | GmbZIP07g-HBT-F | 5' CGCGGATCCATGAACTTCAGGAACTTTGGTG 3' | BamHI |
|  | GmbZIP07g-HBT-R | 5' CGCGGATCCCCATGGTCCAGTAAGTGTCCT 3' | BamHI |
| **RT-PCR** | qGmbZIP07g-F | 5' GACAAGCACAACAGCACCAACA 3' | no |
|  | qGmbZIP07g-R | 5' TCAGCAACTCCAATCAACCCTCT 3' | no |
|  | qGmbZIP04g-F | 5' GAACATCCAATCAGCAGCCAC 3' | no |
|  | qGmbZIP04g-R | 5' AACCATACCAATCCCTCCACCT 3' | no |
|  | GmRCAα-F | 5' GATGGGCGTATGGAGAAGTTCT 3' | no |
|  | GmRCAα-R | 5' TGCGGAAAATTCCATTGCA 3' | no |
|  | GmRCAα-P | 5' ACGATCGTGTTGGCG 3' | no |
|  | Tubulin-F | 5' GAGAGCAACATGAACGACCTTGT 3' | no |
|  | Tubulin-R | 5' TCCTCATACTCCCCATCATCCT 3' | no |
|  | Tubulin-P | 5' TCAGAGTACCAGCAGTACCAGGATGCCA 3' | no |

| **Supplemental Table S2** Y1H screen results | | |  |  |
| --- | --- | --- | --- | --- |
| Gene symbol/  locus | clone numbers | Gene description |  |  |
| Glyma02g14880 | 6 | Glycine max stress-related protein 1 (AREB1) |  |  |
| Glyma07g33600 | 6 | ABSCISIC ACID-INSENSITIVE 5-like protein 7-like |  |  |
| Glyma04g04170 | 3 | ABSCISIC ACID-INSENSITIVE 5-like protein 5-like |  |  |
| Glyma06g04353 | 2 | transcription factor bZIP71 |  |  |
| LOC100800095 | 2 | zinc finger protein ZAT10-like |  |  |
| LOC100778467 | 2 | zinc finger protein ZAT11-like |  |  |
| LOC100810972 | 2 | lysine-specific demethylase REF6-like |  |  |
| LOC100775271 | 2 | lysine-specific demethylase REF6-like |  |  |
| LOC100810437 | 3 | lysine-specific demethylase REF6-like |  |  |
| LOC100786999 | 1 | transcription factor HB29-like |  |  |
| LOC100808910 | 1 | ZF-HD homeobox protein At4g24660-like |  |  |
| LOC102660309 | 1 | transcription factor HB29-like |  |  |
| LOC100812599 | 1 | trihelix transcription factor GTL1-like |  |  |
| LOC100811234 | 1 | heat shock protein 83-like |  |  |
| 100170750 | 1 | aluminum-activated citrate transporter |  |  |
| Glyma04g19030 | 3 | heme oxygenase 1 |  |  |
| LOC100780624 | 6 | heavy metal-associated isoprenylated plant protein 26-like |  |  |
| LOC100801213 | 2 | phosphatidylinositol:ceramide inositolphosphotransferase 1-like | |  |
| LOC100815011 | 2 | phosphatidylinositol:ceramide inositolphosphotransferase 1-like | |  |
| LOC100797212 | 2 | phosphatidylinositol:ceramide inositolphosphotransferase 1-like | |  |
| 547511 | 1 | Glycine max catalase (CAT4) |  |  |
| LOC100807355 | 1 | photosystem II 22 kDa protein, chloroplastic-like |  |  |
| LOC100816781 | 1 | putative DNA-binding protein ESCAROLA-like |  |  |
| LOC100818917 | 1 | non-specific lipid-transfer protein-like |  |  |
| LOC100794944 | 1 | chlorophyll a-b binding protein 7, chloroplastic-like |  |  |
| LOC100802138 | 1 | chlorophyll a-b binding protein 7, chloroplastic-like |  |  |
| LOC100820030 | 1 | NAD kinase 2, chloroplastic-like |  |  |
| LOC100811855 | 1 | CBS domain-containing protein CBSX5-like |  |  |
| LOC100789241 | 1 | mitogen-activated protein kinase kinase 2-like |  |  |
| LOC100800878 | 1 | mitogen-activated protein kinase 19-like |  |  |
| LOC100811719 | 1 | P34 probable thiol protease-like |  |  |
| LOC100170730 | 1 | peroxisomal glycolate oxidase |  |  |
| LOC100794277 | 1 | ribulose bisphosphate carboxylase small chain 1, chloroplastic-like | |  |
| LOC547759 | 1 | thioredoxin |  |  |
| 548082 | 1 | glutamate--ammonia ligase |  |  |
| LOC100527509 | 1 | uncharacterized |  |  |
| LOC100305752 | 1 | uncharacterized |  |  |
| LOC100306491 | 1 | uncharacterized | | |
| LOC100499794 | 1 | uncharacterized | |  |
| LOC100817892 | 1 | uncharacterized |  |  |
| LOC100782176 | 1 | uncharacterized | | |
| LOC100785302 | 1 | uncharacterized |  |  |
| LOC100779195 | 1 | uncharacterized |  |  |
| LOC100817903 | 1 | uncharacterized |  |  |
| LOC100500127 | 1 | uncharacterized |  |  |

**Supplemental Table S3** The LUC, REN and LUC/REN values in *Arabidopsis* protoplasts.

| Sample | LUC | REN | LUC/REN |
| --- | --- | --- | --- |
| GmRCAα_pro_::LUC+EV+pPTRL | 5874 | 9203 | 0.6382701 |
| GmRCAα_pro_::LUC+EV+pPTRL | 8005 | 10188 | 0.7857283 |
| GmRCAα_pro_::LUC+EV+pPTRL | 5745 | 9501 | 0.6046732 |
| GmRCAα_pro_::LUC+EV+pPTRL | 8200 | 10503 | 0.7807293 |
| GmRCAα_pro_::LUC+GmbZIP04g+pPTRL | 1196 | 563 | 2.1243339 |
| GmRCAα_pro_::LUC+GmbZIP04g+pPTRL | 1035 | 502 | 2.061753 |
| GmRCAα_pro_::LUC+GmbZIP04g+pPTRL | 1490 | 545 | 2.733945 |
| GmRCAα_pro_::LUC+GmbZIP04g+pPTRL | 1603 | 606 | 2.6452145 |
| GmRCAα_pro_::LUC+GmbZIP07g+pPTRL | 3440 | 2483 | 1.3854209 |
| GmRCAα_pro_::LUC+GmbZIP07g+pPTRL | 3134 | 2566 | 1.2213562 |
| GmRCAα_pro_::LUC+GmbZIP07g+pPTRL | 4083 | 2822 | 1.4468462 |
| GmRCAα_pro_::LUC+GmbZIP07g+pPTRL | 5869 | 3169 | 1.8520038 |

EV indicates the empty factor, HBT95::sGFP(S65T)-NOS.

The pPTRL vector was used as an internal control to normalize values obtained after each transformation.

The GmbZIP04g and GmbZIP07g indicate the recombination vectors that GmbZIP04g and GmbZIP07g were linked after the HBT95 promoter.

**Figure legends**

**Supplemental Figure. S1** Relative reporter activity (LUC/REN) with different effectors (EV, GmbZIP04g and GmbZIP07g) expression in *Arabidopsis* protoplasts. EV indicates empty vector, HBT95::sGFP(S65T)-NOS. To normalize values obtained after each transformation, a *Renilla* luciferase gene was used as an internal control. The *GmRCAα_pro_::LUC* and the *Renilla* luciferase construct were co-transformed with the effector EV, GmbZIP04g or GmbZIP07g. The error bars are based on four replicates. The comparisons were performed using one-way ANOVA, and p values are 6.99×10^-6^ and 0.002218, respectively. ** denotes significant difference at P= 0.01.
